# A Triterpenoid from *Thalictrum fortunei* Induces Apoptosis in BEL-7402 Cells Through the P53-Induced Apoptosis Pathway

**DOI:** 10.3390/molecules16119505

**Published:** 2011-11-15

**Authors:** Xiantao Zhang, Ming Zhao, Lvyi Chen, Haoyan Jiao, Hongxia Liu, Liyan Wang, Shuwei Ma

**Affiliations:** 1 Guangdong Research Institute of Traditional Chinese Medicine, Guangzhou 510520, China; 2 Department of Pharmacy Engineering, Institute of Chemistry and Chemical Engineering, Qiqihar University, Qiqihar 161006, China; 3 School of Pharmacy, South-Central University for Nationalities, Wuhan 430074, China

**Keywords:** *Thalictrum fortunei*, anti-tumor, triterpenoid, Bel-7402 cells

## Abstract

*Thalictrum fortunei* S. Moore, a perennial plant distributed in the southeastern part of China, has been used in Traditional Chinese Medicine for thousands of years for its antitumor, antibacterial and immunoregulatory effects. In order to investigate the active components and the mechanism of the anti-tumor effects of *Thalictrum fortunei*, the growth inhibitory effects of eight triterpenoids isolated from the aerial parts of the plant on tumor cell lines were examined by 3-(4,5)-dimethylthiazoy1-3,5-diphenyltetrazolium bromide (MTT) assay. The MTT-assay results showed that the inhibitory activity of 3-*O-β*-d-glucopyranosyl-(1→4)-*β*-d-fucopyranosyl(22*S*,24*Z*)-cycloart-24-en-3*β*,22,26-triol 26-*O-β*-d-glucopyranoside (**1**) was stronger than that of the other seven tested triterpenoids on human hepatoma Bel-7402 cell line (Bel-7402), human colon lovo cells (LoVo), human non-small cells lung cancer NCIH-460 cells (NCIH-460) and human gastric carcinoma SGC-7901 cells (SGC-7901) after 48 h treatment *in vitro*, with the IC_50_ values of 66.4, 84.8, 73.5, 89.6 μM, respectively. Moreover, the antitumor mechanism of compound **1** on Bel-7402 cell was explored through nucleus dyeing, fluorescence assay, flow cytometry and western blot. The flow cytometric analysis results revealed that compound **1** caused apoptosis and mitochondrial membrane potential (MMP) loss in Bel-7402 cells. A fluorescence assay indicated that intracellular reactive oxygen species (ROS) were markedly provoked by compound **1** treatment compared to control cells. Immunoblot results showed that compound **1** significantly increased the expression levels of cleaved caspase-3, P53 and Bax protein, and decreased the expression level of Bcl-2 protein. These findings indicate that compound **1** inhibits the growth activity of tumor cells, probably through the P53 protein-induced apoptosis pathway.

## 1. Introduction

Hepatocellular carcinoma is the eighth most common cancer over the World by number of cases and the third most common cause of death from cancer due to its very poor prognosis. Eighty-two percent of cases are in developing countries, with fifty-five percent being in China [1]. Up to now, chemotherapy has provided significant survival benefits for patients with hepatocellular carcinoma [2], however, it is associated with significant normal tissue toxicity. Thus, it is necessary to find new effective medications for hepatocellular carcinoma. Development of pharmacologically effective agents with little toxicity or few side effects from natural products has become a new important research topic in this area.

Previous reports have indicated that antitumor activity was found in the extract of *Thalictrum fortunei* [3]. Additionally, many triterpenoids from *Thalictrum fortune* were isolated and identified [4,5]. Therefore, the aim of this study was to evaluate the cytotoxicity of eight triterpenoids from *Thalictrum fortunei* on SGC-7901 cells, Bel-7402 cell, LoVo cells and NCIH-460 cells. Further, a representative compound was chose to investigate its anti-tumor mechanism on BEL-7402 cells [6]. To study of the mechanism of cytotoxicity, cell nucleus morphology was observed using Hoechst 33258 staining, and apoptosis were analyzed by flow cytometry. To further elucidate the apoptosis pathway, the mitochondrial membrane potential (MMP), reactive oxygen species (ROS), caspase-3, P53, Bcl-2 and Bax protein expression were also assayed.

## 2. Results and Discussion

### 2.1. Compounds Isolated from *Thalictrum fortunei*

**Figure 1:**
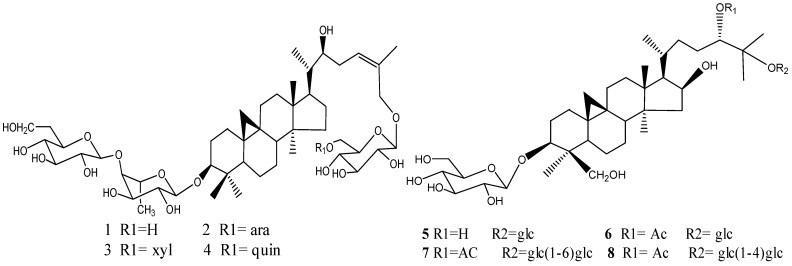
Structures of eight triterpenoids identified from *Thalictrum fortunei*.

Eight glycosides were isolated from the aerial parts of *Thalictrum fortunei*. The chemical structures were elucidated as 3-*O-β*-d-glucopyranosyl-(1→4)-*β*-d-fucopyranosyl(22*S*,24*Z*)-cycloart-24-en-3*β*,22,26-triol 26-*O-β*-d-glucopyranoside (**1**), 3-*O-β*-d-glucopyranosyl-(1→4)-*β*-d-fucopyranosyl(22*S*,24*Z*)-cycloart-24-en-3*β*,22,26-triol 26-*O-α*-L-arabinopyranosyl-(1→6)-*β*-d-glucopyranoside (**2**), 3-*O-β*-d-glucopyranosyl-(1→4)-*β*-d-fucopyranosyl(22*S*,24*Z*)-cycloart-24-en-3*β*,22,26-triol 26-*O-β*-d-xylopyranosyl-(1→6)-*β*-d-glucopyranoside (**3**), and 3-*O-β*-d-glucopyranosyl-(1→4)*-β*-d-fucopyranosyl(22*S*,24*Z*)-cycloart-24-en-3*β*,22,26-triol 26-*O-β*-d-quinovopyranosyl-(1→6)-*β-*d-glucopyranoside (**4**), (24*S*)-cycloartane-3*β*,16*β*,24,25,30-pentaol 3,25-di-*O-β*-d-glucopyranoside (**5**), 24-*O*-acetyl-(24*S*)-cycloartane-3*β*,16*β*,24,25,30-pentaol 3,25-di-*O-β*-d-glucopyranoside (**6**), 3-*O-β-D*-glucopyranosyl-24-*O*-acetyl-(24S)-cycloartane-3*β*,16*β*,24,25,30-pentaol 25-*O*-*β-D*-glucopyranosyl(1→6)-*β-D*-glucopyranoside (**7**) and 3-*O-β*-d-glucopyranosyl-(24*S*)-cyclo-artane-3*β*,16*β*,24,25,30-pentaol 25-*O-β*-d-glucopyranosyl(1→4)*-β*-d-glucopyranoside (**8**) (shown in Figure 1), by extensive NMR methods, HRESIMS, and hydrolysis respectively. Their purities were all proven to be higher than 98% by HPLC analysis.

### 2.2. Growth Inhibitory Effect of Eight Triterpenoids on SGC-7901, Bel-7402, Lovo and NCIH-460 Cells

The MTT assay was used to determine the cell viability of HSGC-7901, Bel-7402, lovo and NCIH-460 cells after treatment with the different triterpenoids. As shown in Table 1, compound **1** exhibited the strongest *in vitro* suppressive effect on all the above tumor cells at 48 h after treatment, with IC_50_ values of 66.4, 84.8, 73.5, 89.6 μM, respectively, while compounds **2–4** showed milder cytotoxic effects than compound **1** and compounds **5–8** displayed the lower cytotoxic effects among the eight triterpenoids on these four tumor cell lines.

### 2.3. Effect of Compound ***1*** on the Viability of Bel-7402 Cells

**Figure 2:**
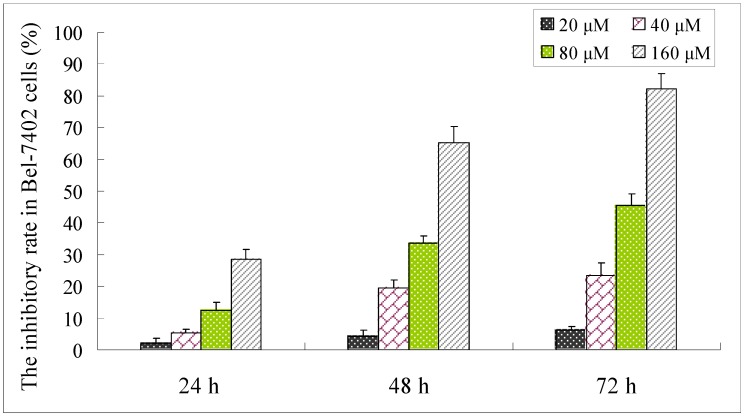
The inhibitory rate of human hepatoma carcinoma cells (Bel-7402) after being treated with compound **1** at different concentrations (20, 40, 80, 160 μM) for 24 h, 48 h and 72 h, respectively. Date were shown as means ± S.D. (n = 6). The data are represented as mean ± S.D for six independent experiments.

Human hepatoma BEL-7402 cell line was selected as the model strain for cytotoxicity assessment [7,8,9], and to elucidate the mechanisms of the cytotoxicity because it showed the strongest cytotoxity than other compounds in this cell line, as shown in Table 1. We evaluated the inhibitory effect of compound **1** on Bel-7402 cells by the MTT assay. The inhibition ratio of compound **1** in tumor cells at different concentrations (20, 40, 80, 160 μM) for 24 h, 48 h and 72 h is shown in Figure 2. Compound **1** exhibited a dose and time-dependent suppressive effect on the growth of Bel-7402 cells. Hence, the possible mechanism of the anti-tumor effects of the triterpenoid on other tumor cells also become an essential objective of our research.

### 2.4. Effect of Compound ***1*** on the Morphological Changes of Bel-7402 Cells

**Figure 3:**
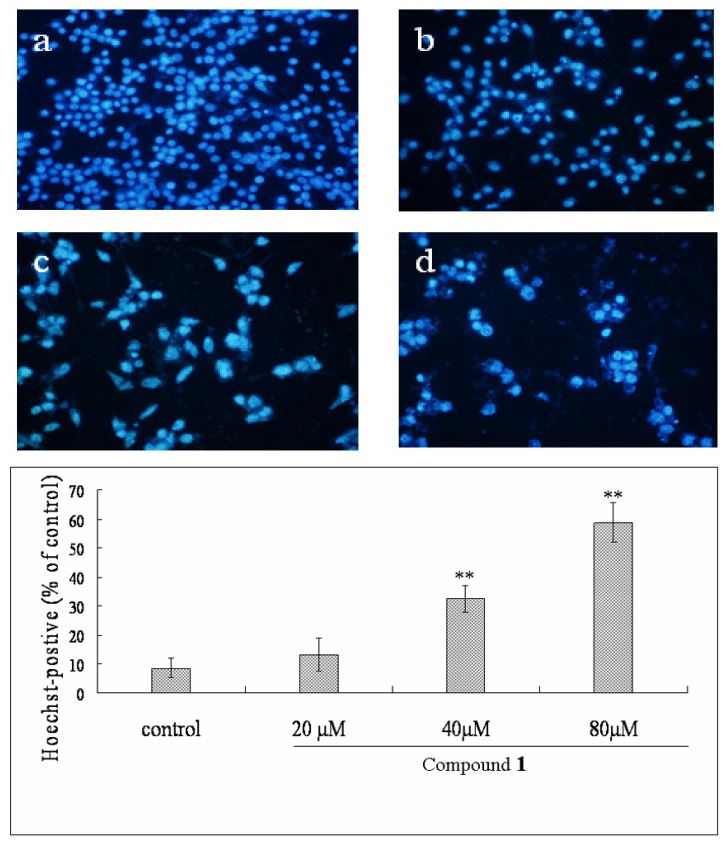
Compound **1** induced apoptosis of Bel-7402 cells for 48 h. Effect of compound **1** on Bel-7402 cells morphological change by hoechst 33258 staining (×200): (**a**) Control cells proliferated normally and cells appeared enlarged with prominent nuclei; (**b-d**) The addition of compound **1** (20, 40 and 80 μM) to the cultures of Bel-7402 cells showed dose-dependent inhibition of cell growth, the nuclei exhibited bright condensed chromatin. Compound **1** led to individual cell to shrink and separate from consecutive cells.

Hoechst 33258 staining was applied to investigate whether Bel-7402 cells underwent cell death via apoptosis or necrosis. As shown in Figure 3, after administration of different concentrations of compound **1**, the Bel-7402 cells revealed marked nuclear condensation, membrane blebbing, nuclear fragmentation and apoptotic bodies, all of which are characteristic of apoptotic programmed cell death. After treatment with compound **1** at the concentrations of 20, 40, 80 μM for 48 h, Hoechst-postive cells were increased from 13.4% to 32.5% and 58.7%, respectively. In contrast, those characteristics were not displayed in control cells.

### 2.5. Flow Cytometry Analysis of Compound ***1***-Induced Bel-7402 Apoptosis

**Figure 4:**
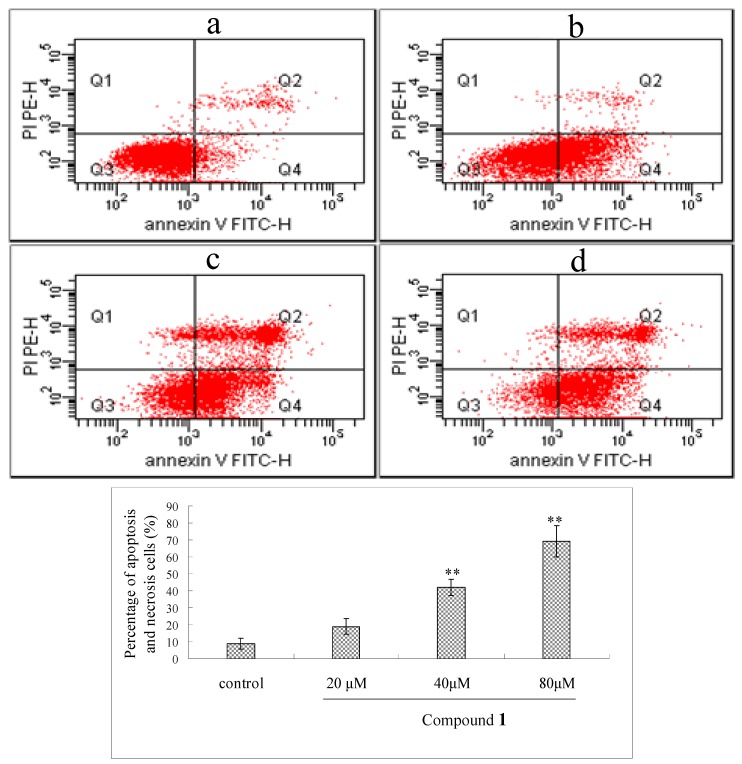
Flow cytometric analysis of Bel-7402 cells apoptosis following exposed to compound **1.** Annexin V/PI double-staining assay of Bel-7402 cells. X axis shows FITC-labeled Annexin V positive cells and Y axis shows PI labeled population. The percentage of those four sections cells was indicated. (**a**) control, cells were not treated with compound **1**; (**b-d**) cells were treated with compound **1** at concentrations of 20, 40 and 80 μM, respectively. The data are represented as mean ± S.D for six independent experiments. * P < 0.05 and ** P < 0.01 vs. control.

Annexin V/PI staining assay was used to further confirm the apoptosis induced by compound **1**. As shown in Figure 4, after treated with compound **1** at the concentrations of 20, 40, 80 μM for 48 h, the early and median apoptotic cells (right low section of fluorocytogram) were increased strikingly (from 19.3% to 29.6% and 53.4%, respectively) and the late apoptotic and necrotic cells (right upper section of fluorocytogram) were also changed (from 6.3% to 6.4% and 24.6%). These results suggested that apoptosis induction by compound **1** was involved in its antitumor effects.

### 2.6. Effect of Compound ***1*** on MMP and ROS Levels

**Figure 5:**
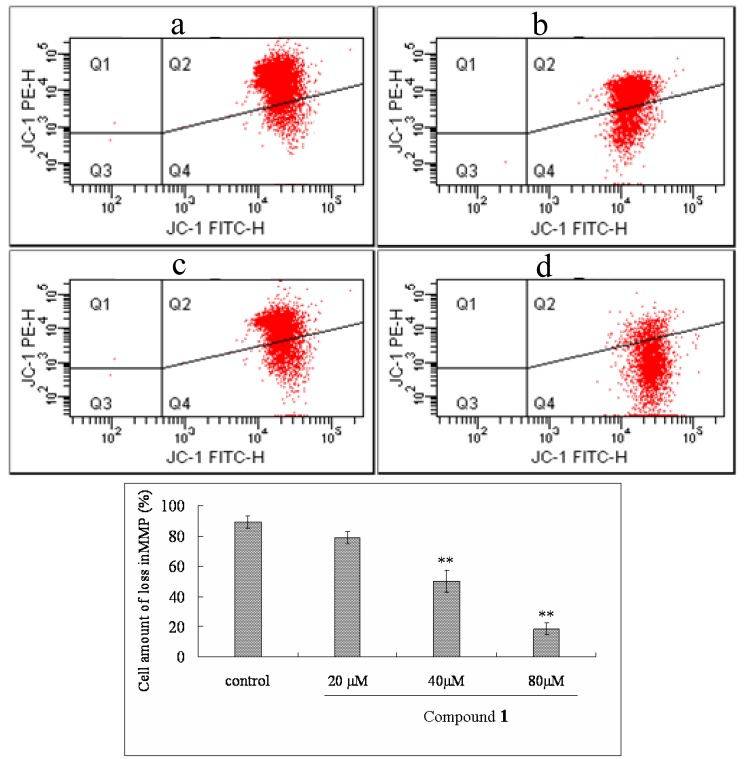
Mitochondria membrane potential of Bel-7402 cells were determined by flow cytometric analysis following exposed to compound **1** for 48 h. Q2 district showed MMP normal cells and Q4 district demonstrated MMP loss cells. (**a**) Control, cells were not treated with compound **1**; (**b-d**) cells were treated with compound **1** at concentrations of 20, 40 and 80 μM, respectively. The data are represented as mean ± S.D for three independent experiments. * P < 0.05 and ** P < 0.01 vs. control.

**Figure 6:**
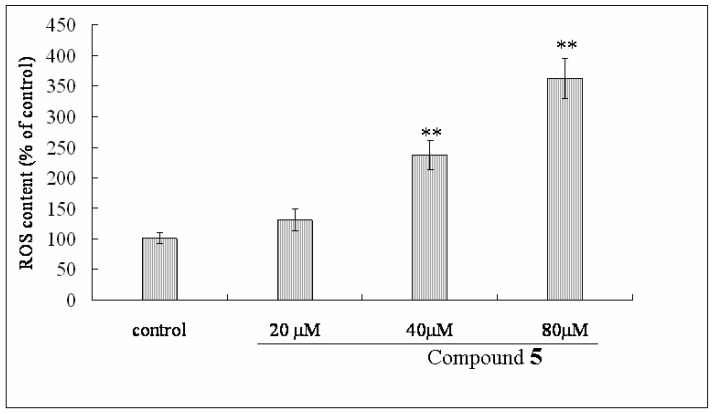
After the treatment of Bel-7402 cells with different concentrations of compound **1** for 48 h, ROS production were assayed by the fluorescence intensity of DCF with the enzyme-immunoassay instrument at an excitation wavelength of 485 nm and an emission wavelength of 535 nm. The data were represented as mean ± standard S.D for six independent experiments. * P < 0.05 and ** P < 0.01 vs. control.

To investigate the alteration of mitochondrial membrane potential (MMP) in Bel-7402 cells treated with compound **1**, the fluorescent probe JC-1 was used to determine this parameter. As shown in Figure 5, treatment with different concentrations of compound **1** (at 20, 40, 80 μM) for 48 h induced a significant loss of MMP from 78.8% to 50.3% and 18.6% in a dose-dependent manner. Moreover, to assess the effect of compound **1** on intracellular ROS, after Bel-7402 cells were incubated with concentrations of compound **1** (at 20, 40, 80 μM) for 48 h, intracellular ROS content was significantly increased from 130.45% to 237.3% and 362.7% (Figure 6) in a dose-dependent manner.

Mitochondria are considered to play an important role in apoptosis, the the mitochondrial apoptosis pathway has been described as an important signaling pathway of apoptosis [10,11]. Mitochondrial membrane potential change constitutes an early event in the apoptotic process, which leads to the release of apoptosomes from mitochondria. Further, the apoptosome triggers formation of apoptotic complex and activation of the caspase-9, and leads to the proteolytic activation of caspase-3, the primary caspase of the cell [12].

Loss in MMP induced superabundant ROS, resulting in membrane lipid peroxidation, nitration of proteins, and degradation of DNA, all of which are associated with the course of apoptosis [13]. ROS itself present at high levels also could cause severe damage to DNA, proteins and lipids, which may lead to apoptosis [14]. Our studies indicated that treatment with compound **1** induced increased ROS levels and loss in MMP, both of which promote apoptosis through cytochrome c release from mitochondria to the cytosol.

### 2.7. Effect of Compound ***1*** on the Expression of Caspase-3, P53, Bcl-2 and Bax Protein

**Figure 7:**
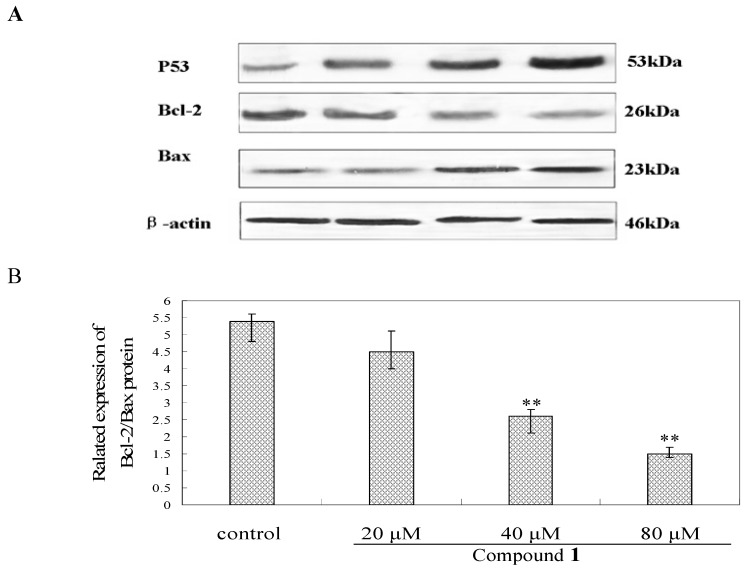
Bel-7402 cells were incubated with compound **1** at the concentrations of 20, 40 and 80 μM for 48 h, respectively, then, the different proteins levels were measured by westblot method. (**A**) Effects of compound **1** on the expression levels of P53, Bcl-2 and Bax proteins; (**B**) The ratio of Bcl-2/Bax protein expression. ** p < 0.01 as compared to control.

As shown in Figure 7, after incubation with compound **1** at the concentration of 20, 40 and 80 μM for 48 h, the expression of caspase-3 and P53 protein increased dramatically. Furthermore, higher dosage of compound **1** on Bel-7402 cells for 48 h resulted in the decreased Bcl-2 protein levels and the increased Bax protein expression, which leads to a decrease of the Bcl-2/Bax ratio.

These cumulative findings confirmed that p53 was involved in several critical pathways including cell cycle arrest, apoptosis, DNA repair, and cellular senescence, which are essential for normal cellular homeostasis and for maintaining genomic integrity [15].

One pathway of apoptosis is that p53 itself translocates to mitochondria, which are considered to play a important role in apoptosis and where it activates the mitochondrial apoptotic pathway by binding to the anti-apoptotic proteins Bcl-2 and Bcl-xL [16]. Another apoptosis pathway, an accumulation of p53 in the cytosol can act in a similar way to BH3-only proteins, thus inducing the oligomerization of Bax [17]. Finally, p53 leads to the expression of pro-apoptotic proteins, including members of the BH3-only group of Bcl2-related proteins such as Bid, which can trigger the mitochondrial apoptotic pathway [18]. The p53 protein induces the transcription of the TIGAR gene, which lowers the intracellular concentrations of fructose-2,6-bisphosphate and thus decreases overall levels of intracellular reactive oxygen species (ROS) [19]. Our result implied that treatment with compound **1** increased the expression of P53 protein, which leading to cell apoptosis through several pathways such as MMP, ROS, Bax and Bcl-2 protein, *etc*.

**Figure 8:**
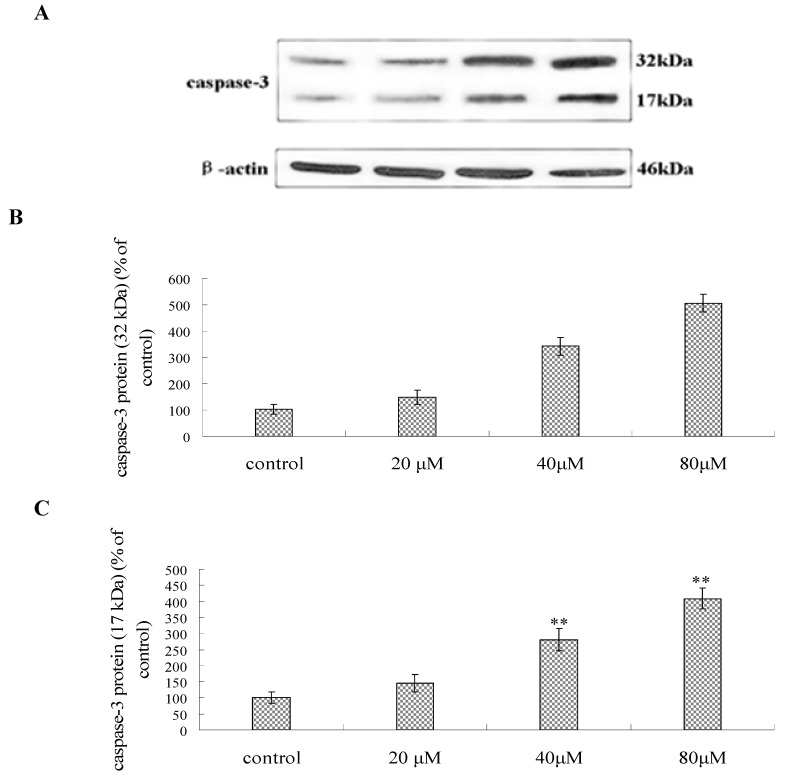
Bel-7402 cells were incubated with compound **1** at the concentrations of 20, 40 and 80 μM for 48 h, respectively, then, the caspasse-3 (32 kDa) proteins and the cleavage product (17 kDa) of caspase-3 protein were measured by westblot method. (**A**) Effects of compound **1** on the expression levels of caspase-3 proteins. (**B**) The bar showed the caspasse-3 (32 kDa) proteins expression. (**C**) The bar displayed the cleavage product (17 kDa) of caspase-3 protein expression. ** p < 0.01 as compared to control.

Caspases are a family of intracellular protein involved in the initiation and execution of apoptosis [20]. Activation of caspase-3 was also confirmed by Western blot. As shown in Figure 8, the protein abundance of caspase-3 (32 kDa) increased in a dose dependent manner. The cleavage product (17 kDa) of caspase-3 was also enhanced in Bel-7402 cells after exposure to compound **1**. The control cells did not show any evidence of increased caspase-3-like activity and the 17 kDa cleavage product, suggesting caspase-3 was specifically activated in Bel-7402 cells after treatment with compound **1**. The results were consistent with the expression levels of antiapoptotic and proapoptotic Bcl-2 family proteins.

## 3. Experimental

### 3.1. Materials

The aerial parts of *Thalictrum fortunei* S. Moore were collected in Wuhu City, Anhui Province, China, in April 2009, and authenticated by Dr. Ming-Jian Qin of China Pharmaceutical University. A voucher specimen (no.090192) was deposited in the herbarium of China Pharmaceutical University, Nanjing. The coarse powder of stems and leaves were air-dried in the shade and stored in a well-closed vessel for use. 3-(4,5-Dimethylthiazol-2-yl)-2,5-diphenyltetrazolium bromide (MTT), 2’,7’-dichloro-flurescein diacetate (DCFH-DA) and 5,5’,6,6’-tetrachloro-1,1’,3,3’-tetraethylbenzimidazolcarbo-cyanine iodide (JC-1) were purchased from Amresco Inc. (Solon, OH, USA). Fetal calf serum was obtained from Si-ji-qing Biotechnology (Hangzhou, China). RPMI Medium 1640 was purchased from Gibco (Paisley, Scotland, UK). Penicillin and streptomycin were obtained from Gibco BRL (Div. of Invitrogen, Gaithersburg, MD, USA). Hoechst 33258, Annexin V-FITC apoptosis detection Kit, P53 anti-body, caspase-3, Bcl-2 and Bax antibody, anti-rabbit and anti-mouse IgG were obtained from Santa Cruz Biotechnology (Santa Cruz, CA, USA). Nitrocellulose membrane and ECL Western detection reagent was purchased from Amersham Bioscience (Piscataway, NJ, USA). All chemical solvents were analytical grade and bought from Merck.

### 3.2. Isolation and Identification of Compounds from Thalictrum fortunei S. Moore. Rhizome

The dried aerial parts (5.2 kg) of *Thalictrum fortunei* were extracted with 95% EtOH (3 × 20 L) under reflux. The EtOH extract was suspended in water and then successively extracted with petroleum ether, EtOAc, and *n*-BuOH. The *n*-BuOH solution was concentrated and given a residue (207 g), which was subjected to repeated chromatography on silica gel column and eluted with chloroform-methanol gradient solvent system, then purified with reversed-phase CC and HPLC. The structure of compound was confirmed by spectrum methods (UV, IR, MS, ^1^H-NMR and ^13^C-NMR).

### 3.3. Cell Lines and Cell Culture

SGC-7901 cells, Bel-7402 Cells, human colon LoVo cells and NCIH-460 cells were obtained from the Animal Experimental Center of Medical College of Sun Yat-Sen University (Guangzhou, China), maintained in RPMI 1640 culture medium plus 10% calf serum and 100 U/mL penicillin, and 100 μg/mL streptomycin in a 37 °C incubator supplied with 95% room air and 5% CO_2_. After 60–80% confluency, the cells were trypsinized with 0.25% trypsin (Amresco, dissolved in PBS, pH 7.4), counted and placed down at needed density for treatment. All cells were prepared to use at 37 °C for 24 hours CO_2_ incubation after sub-cultured into new dishes.

### 3.4. Assessment of Anti-Tumor Activities of Eight Triterpenoids from Thalictrum fortunei by MTT Assay

The MTT assay was used to determine the effect of the eight triterpenoids on overall proliferation of SGC-7901, Bel-7402, lovo and NCIH-460 cells. The activity was measured by the previously described method [21]. Briefly, cells in exponential growth were plated in a 96–well plate and grown to 70–80% confluency, followed treatment with the eight triterpenoids at different concentrations (5, 10, 20, 40, 80, 160 μM) for 48 h, respectively. Then, MTT (100 μL, 5 mg/mL) in PBS was added, and the cells were further incubated at 37 °C for 4 h to allow for complete cleavage of the tetrazolium salt by metabolically active cells. Finally, the supernatant was discarded and dimethyl sulphoxide (DMSO, 150 μL) was added. The 96–well plate was vibrated on a micro-vibrator for an additional 10 min, and the optical density of each well was measured at λ_490nm_ with an enzyme-immunoassay instrument (Thermo Multiskan Ascent). The growth inhibitory ratio of each triterpenoid was calculated based on the following formula:

Inhibitory ratio of growth (%) = (Average O.D. of control group – Average O.D. of treated group) / Average O.D. of control group × 100%.

### 3.5. Assessment of Anti-Tumor Activity of Compound ***1*** on Bel-7402 Cells by MTT Assay

Base on the above MTT-colorimetric assay, the Bel-7402 cells were treated with compound **1** (20, 40, 80, 160 μM) for 24 h, 48 h and 72 h, respectively. MTT (5 mg/mL) in PBS was then added to wells and the cells incubated at 37 °C for 4 h to allow for complete cleavage of the tetrazolium salt by metabolically active cells. Finally, the supernatant was removed and DMSO (150 μL) was added. The 96–well plate was vibrated on micro-vibrator for additional 10 min, and the optical density of each well was measured at λ_490nm_ with the enzyme-immunoassay instrument (Thermo Multiskan Ascent).

### 3.6. Apoptosis Analysis by Hoechst 33258 Staining

Hoechst 33258 staining was carried out as previously described [22]. Briefly, the Bel-7402 cells in logarithmic growth phase were placed down at a final concentration of 1 × 10^5^ cells/well in a 6-well culture plate. A slide was placed on the bottom of each well to allow cells to grow on its surface as a monolayer. After confluence, the cells were exposed to chrysin (20, 40, 80 μM) for 48 h. For Hoechst 33258 staining, the cells were fixed with ice-cold methanol at room temperature for 5 min, washing twice with ice-cold PBS, and then loaded with Hoechst 33258 for additional 20 min. The changes in the nuclei of cells after Hoechst 33258 staining were observed under a fluorescence microscope (Olympus, BX-60 Japan) at 200× magnification for 10 min.

### 3.7. Determination of Cell Apoptosis by Annexin V/PI Double Staining

The Bel-7402 cells were exposed to compound **1** (20, 40, 80 μM) for 48 h. Apoptosis-mediated cell death of tumor cell was examined using a double staining method with an FITC-labeled Annexin V/PI Apoptosis Detection kit (Bestbio, Shanghai) strictly according to the manufacturer’s instructions. Flow cytometric analysis was performed 10-15 min after supravital staining. Data acquisition and analysis were performed in Cell Quest software (Beckman). The left lower section of fluorocytogram (An^−^, PI^−^) showed the normal cells, the right lower section of fluorocytogram (An^−^, PI^−^) displayed the early and median apoptosis cells, and the right upper section of fluorocytogram (An^−^, PI^−^) represented the late apoptosis cells.

### 3.8. Measurement of MMP *by* Flow Cytometry

MMP was detected as described in a previous report [23]. Briefly, after cells were pre-incubated with compound **1** at the different concentration, they were centrifuged for 5 min at 2,000 rpm, and collected at the concentration of 5 × 10^5^, and incubated in Hank’s solution containing 10 μg/mL JC-1 for 30 min at 37 °C, then was centrifuged for 5 min at 2,000 rpm. Finally, cells sample was collected and washed twice with pre-warmed Hank’s solution (37 °C) followed by re-floating and analyzing with flow cytometry with an excitation wavelength of 480 nm and an emission wavelength of 530 nm.

### 3.9. Measurement of Intracellular ROS by Fluorescent Methods

Intracellular ROS was monitored by using the DCFH-DA fluorescent probe [24]. Intracellular H_2_O_2_ or low-molecular-weight peroxides can oxidize DCFH-DA to the highly fluorescent compound dichlorofluorescein (DCF), Bel-7402 cells were harvested after 48 h treatment by compound **1** (20, 40, 80 μM) and disrupted in cell lysis buffer. Cells were incubated with 10 mM DCFH-DA at 37 °C for 30 min, and then washed twice with PBS. Finally the fluorescence intensity of DCF was measured in the enzyme-immunoassay instrument (Thermo Multiskan Ascent) with an excitation wavelength of 485 nm and an emission wavelength of 535 nm.

### 3.10. Western Blot Analysis

Bel-7402 cells were harvested after 48 h treatment by compound **1** (20, 40, 80 μM) and disrupted in cell lysis buffer, and then they were centrifuged at 13,000 g for 10 min. The supernatant was collected and used for further analyses. To analyze P53, caspase-3, Bax and Bcl-2 proteins, the cytosolic fractions were prepared by differential centrifugation using the method described by Sugawara [22]. The protein concentration was determined using the Bradford staining method, after which equal amounts of protein (20 μg) were electrophoresedon 10–15% density SDS-acrylamide gels. Following electrophoresis, the proteins were transferred from the gel to a nitrocellulose membrane using an electric transfer system. Non-specific binding was blocked with 5% skim milk in TBST buffer (5 mM Tris-HCl, pH 7.6, 136 mM NaCl and 0.1% Tween-20) for 1 h. The blots were incubated with antibodies against caspase-3 (1:250), P53 (1:500), Bcl-2 (1:500), Bax (1:1,000), and *β*-actin (1:500) overnight at 4 °C, after which they were washed three times with 1× TBST. The blots were incubated for 1 h at room temperature with a 1:5,000 dilution of horseradish peroxidase-labeled anti-rabbito or anti-mouse IgG. They were washed three times with 1× TBST, after which they were developed using the ECL Western detection reagents.

### 3.11. Statistical Analysis

The significance of the differences between the different groups was determined with the Student’s t-test. The results are presented as mean ± S.D. of three or six independent experiments. The differences were considered significant at *p < 0.05*.

## 4. Conclusions

In conclusion, our studies demonstrate for the first time that eight triterpenoids isolated from *Thalictrum fortunei* show different potent growth inhibitory activities *in vitro* on several tumor cell lines. Compound **1** induces apoptosis by up-regulating the expressions of caspase-3, P53 and Bax proteins and down-regulating the expression of Bcl-2 protein in Bel-7402 cells, which may contribute to the anti-tumor effects of *Thalictrum fortunei*. These findings provide a relevant basis for developing triterpenoids from *Thalictrum fortunei* as an lead compound for cancer medical treatment.

## Figures and Tables

**Table 1:** Cell growth inhibitory activities of eight triterpenoids in Bel-7402 cells, loVo cells, NCIH-460 cells and SGC-7901 cells.

Compound	Cell line IC_50_ (μM) ^a^			
	Bel-7402	loVo	NCIH-460	SGC-7901
1	66.4	84.8 **	73.5 *	89.6 **
2	89.5	97.5	81.4	96.3
3	83.7	107.3	79.3	112.6
4	83.1	79.4	89.3	98.6
5	150.7	123.6	148.9	166.4
6	162.6	156.8	147.2	169.1
7	162.7	145.9	153.5	157.3
8	149.6	152.1	142.4	152.5

^a^ IC_50_, the concentration that inhibits cell growth by 50%. * P < 0.05 and ** P < 0.01 vs. compound **1** in BEL-7402 cells.
